# Thioredoxin Ameliorates Cutaneous Inflammation by Regulating the Epithelial Production and Release of Pro-Inflammatory Cytokines

**DOI:** 10.3389/fimmu.2013.00269

**Published:** 2013-09-09

**Authors:** Hai Tian, Yoshiyuki Matsuo, Atsushi Fukunaga, Ryusuke Ono, Chikako Nishigori, Junji Yodoi

**Affiliations:** ^1^Redox Bio Science Inc, Kyoto, Japan; ^2^Laboratory of Infection and Prevention, Department of Biological Response, Institute for Virus Research, Kyoto University, Kyoto, Japan; ^3^Division of Dermatology, Department of Internal Related, Kobe University Graduate School of Medicine, Kobe, Japan

**Keywords:** thiroredoxin, topical application, cytokines, keratinocytes, phorbol 12-myristate 13-acetate, cutaneous inflammatory disorders, redox

## Abstract

Human thioredoxin-1 (TRX) is a 12-kDa protein with redox-active dithiol in the active site -Cys-Gly-Pro-Cys-. It has been demonstrated that systemic administration and transgenic overexpression of TRX ameliorate inflammation in various animal models, but its anti-inflammatory mechanism is not well characterized. We investigated the anti-inflammatory effects of topically applied recombinant human TRX (rhTRX) in a murine irritant contact dermatitis (ICD) induced by croton oil. Topically applied rhTRX was distributed only in the skin tissues under both non-inflammatory and inflammatory conditions, and significantly suppressed the inflammatory response by inhibiting the production of cytokines and chemokines, such as TNF-α, Il-1β, IL-6, CXCL-1, and MCP-1. In an *in vitro* study, rhTRX also significantly inhibited the formation of cytokines and chemokines produced by keratinocytes after exposure to croton oil and phorbol 12-myristate 13-acetate. These results indicate that TRX prevents skin inflammation via the inhibition of local formation of inflammatory cytokines and chemokines. As a promising new approach, local application of TRX may be useful for the treatment of various skin and mucosal inflammatory disorders.

## Introduction

Thioredoxin-1 (TRX), a small (12-kDa) protein with a highly conserved redox-active dithiol/disulfide in the active site sequence Cys32-Gly-Pro-Cys35, plays a variety of redox-related roles in essentially all organisms on the earth ranging from *Escherichia coli* to humans (1). TRX catalyzes reduction of disulfide bonds and quenches reactive oxygen species (ROS) by coupling with TRX-dependent peroxidases, or peroxiredoxins. In addition to its anti-oxidant properties, TRX has a crucial role in the redox regulation of cellular signaling and activation. TRX is involved in various redox-dependent cellular processes, such as gene expression, signal transduction, cell growth, and apoptosis, interacting with various kinds of target molecules. Secreted TRX has first been identified as adult T cell leukemia-derived factor produced by human T-lymphotropic virus type I (HTLV-I)-transformed T cells (2). Under stress conditions TRX is released into the extracellular space, where it exerts the cytoprotective effect and cytokine-like activities (3). Transgenic overexpression of TRX and the systemic administration of recombinant human thioredoxin (rhTRX) are effective in a wide variety of inflammatory disease models, such as viral pneumonia, acute lung injury, pancreatitis, myocarditis, chronic obstructive pulmonary diseases, and indomethacin-induced gastric injury (4–9).

Indeed, recent reports have demonstrated that allergic contact dermatitis (ACD), the irritant contact dermatitis (ICD) to croton oil and ultraviolet light-induced dermatitis were unequivocally suppressed in TRX-transgenic mice (10, 11). Intriguingly, overproduction of TRX in these mice did not affect the contact hypersensitivity response in the induction phase of ACD, whereas skin inflammation was suppressed in TRX-transgenic mice after elicitation challenge with DNFB (10). Similarly, exogenously administered TRX exhibited anti-inflammatory activity in the effector phase, but not in the sensitizing phase of ACD. Our previous report has also shown that there were no apparent differences in immune cell populations between TRX-transgenic and wild-type animals, suggesting that the suppression of allergic reaction and inflammation observed in TRX-transgenic mice may not depend on Th1/Th2 polarization or systemic immunosuppression (12). These findings indicate that the anti-inflammatory mechanism of TRX is apparently different from the mechanisms associated with anti-inflammatory agent such as glucocorticoids, which regulate the inflammatory reaction in association with the suppression of immune responses. Analyses of TRX-transgenic mice have strongly suggested possible therapeutic utility of TRX for inflammatory disorders.

Accumulating evidence indicates that epidermal keratinocytes play a major role in the initiation of the inflammatory response in the skin under pathological conditions. Keratinocytes constitute 90% of epidermal cells in the outermost layer of the skin, forming a barrier against the external stimuli and harmful environmental agents. Wilmer et al. (13) reported that cultured keratinocytes produced inflammatory cytokines in response to primary contact irritants including croton oil, and the expression patterns of cytokines correlated with the onset of skin inflammation. Since excessive production of inflammatory cytokines would be the major cause of tissue damages during inflammation, keratinocytes have been implicated as the primary source of inflammatory mediators, contributing to the development of skin inflammation.

In this study, the anti-inflammatory effect of topically applied rhTRX was tested in the well-established model of ICD induced by croton oil (14, 15).

## Results

### Topical application of rhTRX suppresses ICD induced by croton oil

**Figure 1:**
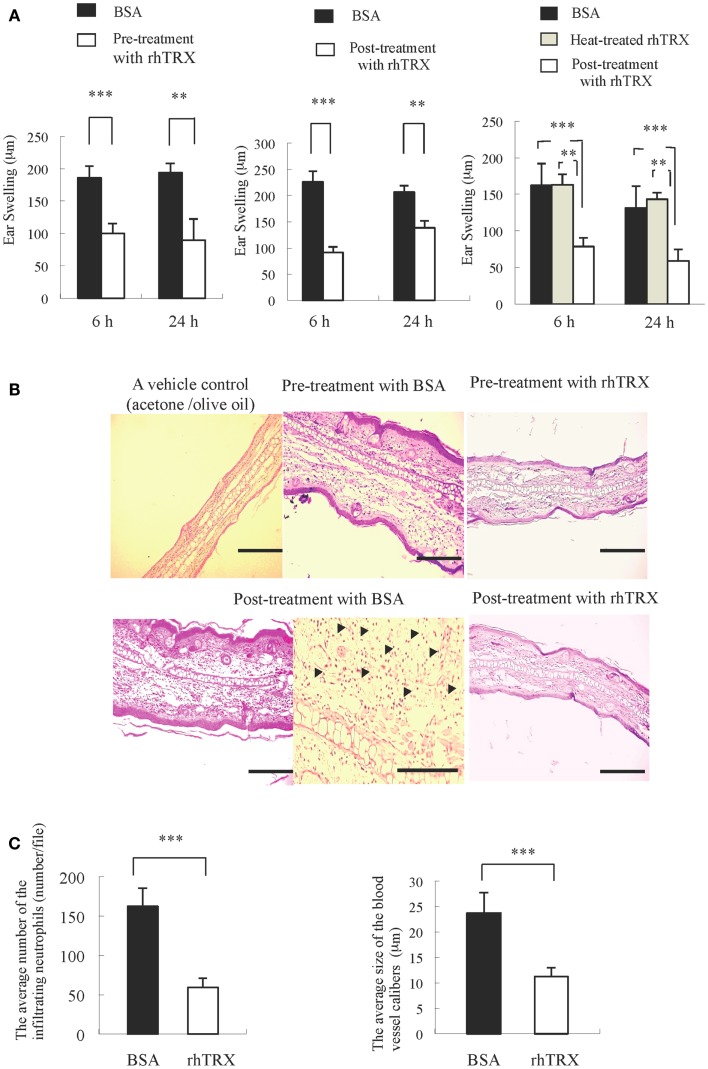
**The topical application of rhTRX strongly suppressed ICD**. **(A)** Inflammatory reactions were expressed as the average increase in ear swelling. Both pre- and post-treatment with rhTRX significantly suppressed ear swelling at 6 and 24 h after croton oil was applied (***P < 0.001, **P < 0.01). Values are the mean SD of each group (Student *t*-test). Left, Middle: *n* = 5, Right: *n* = 3. **(B)** Representative pictures of hematoxylin and eosin staining of the ears. The infiltrating neutrophil and edema were suppressed in both pre- and post-treatment in the rhTRX topical application group, compared with the control group. Acetone/olive oil was used as a vehicle control. Bars, 50 μm. The infiltrating neutrophils were indicated by arrows (Bar, 100 μm). **(C)** The average number of infiltrating neutrophil in the dermis and the dilatation of the capillary blood vessels were significantly reduced in the post-treatment rhTRX group, compared with the control group (***P < 0.001). Data are expressed as the mean ± SD of five mice per group (Student *t*-test).

Croton oil was applied to both sides of murine ear to induce ICD. In order to examine the effect of topically applied rhTRX on ICD, the mice were divided into five groups: (1) a group of mice received rhTRX 2 h before croton oil application (pre-treatment), (2) a group of mice received bovine serum albumin (BSA) 2 h before croton oil application, (3) a group of mice received rhTRX treatment immediately after croton oil application (post-treatment), (4) a group of mice received BSA immediately after croton oil treatment, (5) a group of mice received heat-inactivated rhTRX immediately after croton oil treatment. The degree of ear swelling was measured 6 and 24 h after croton oil application. As shown in Figure 1A, both pre- and post-treatment with rhTRX significantly suppressed the ear swelling induced by croton oil, compared to the BSA group (***P < 0.001, Figure 1A, Left and Middle). Heat-inactivated rhTRX offered no protection against skin inflammation (**P < 0.01, Right). In addition, histological evidence of skin inflammation, such as edema and infiltration of inflammatory cells including neutrophils and macrophages, was suppressed by the topical application of rhTRX (Figure 1B). The number of the infiltrating neutrophils and the caliber of the capillary blood vessels in the dermis were counted and measured for quantitative analysis to evaluate the anti-inflammatory effect of TRX. The results showed that the average number of infiltrating neutrophils and the dilatation of the capillary blood vessels were significantly decreased in the rhTRX treated group, compared to BSA treated group (***P < 0.001, ***P < 0.001) (Figure 1C). These results indicated that topically applied rhTRX suppressed ICD, but rhTRX lost the anti-inflammatory effect by the heat treatment.

### The expression of TNF-α, IL-1β, IL-6, CXCL-1, MIF, and MCP-1 were suppressed by rhTRX in ICD induced by croton oil

**Figure 2:**
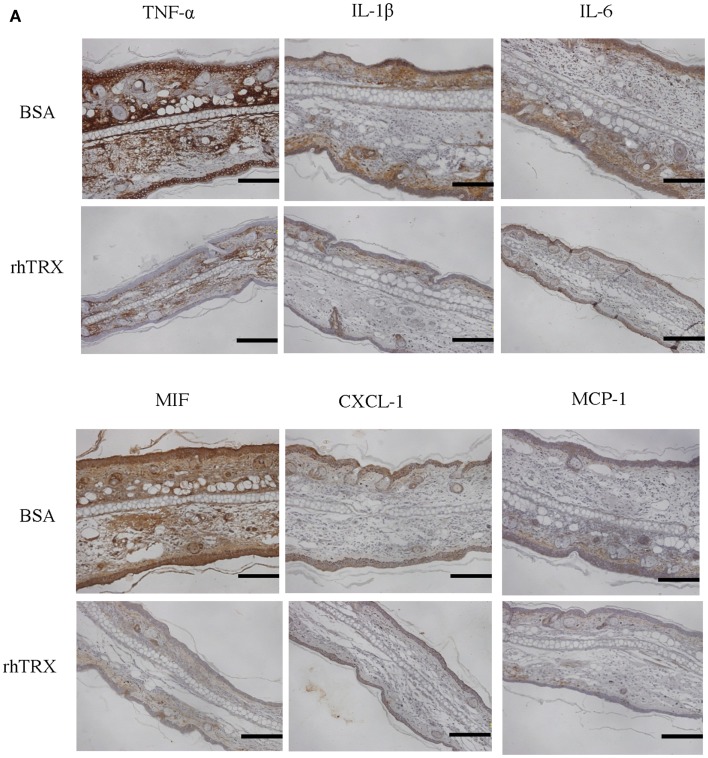

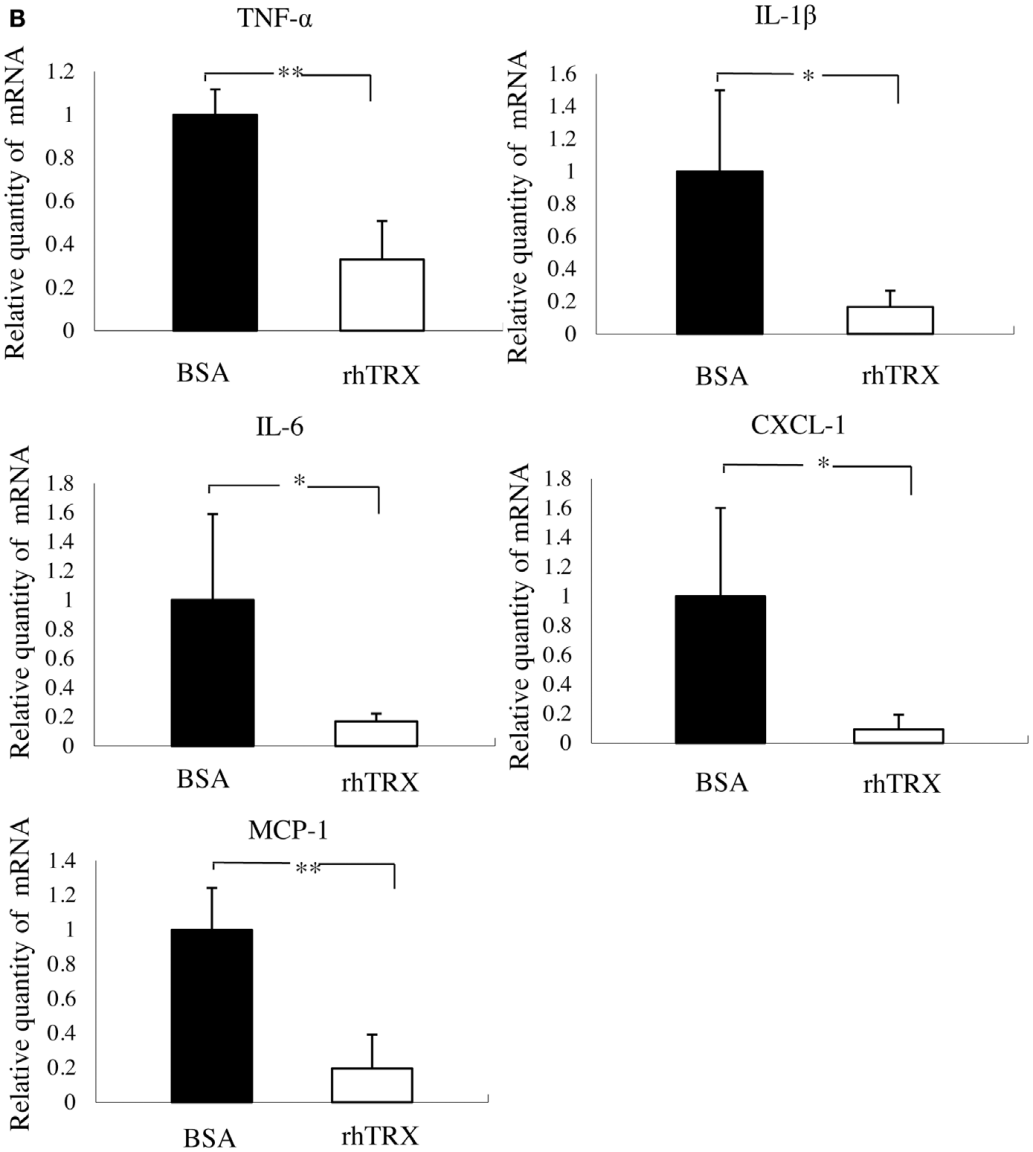
**The topical application of rhTRX suppressed the expression of TNF-α, IL-1β, IL-6, CXCL-1, MIF, and MCP-1 in the ICD model induced by croton oil**. **(A)** Immunohistochemistry and real-time RT-PCR were performed to detect the effect of rhTRX on the production of the cytokines. The results of imunohistochemical staining show the expression of the cytokines. The expression of TNF-α, IL-1β, IL-6, IL-8, MIF, and MCP-1 was strongly suppressed by the topical application of rhTRX. Bars, 50 μm. **(B)** Induction of TNF-α, IL-1β, IL-6, CXCL-1, and MCP-1 were verified by real-time RT-PCR. The mRNA expression was normalized to GAPDH. The mRNA expression levels were determined relative to control sample from BSA treatment group. The mRNA expression of TNF-α, IL-1β, IL-6, CXCL-1, and MCP-1 was significantly suppressed by the topical application of rhTRX (*P < 0.05, **P < 0.01). Values are mean ± SD of five mice per group (Student *t*-test, Wilcoxon signed-rank test).

We investigated the effect of exogenous TRX on the cytokine production in the croton oil-induced ICD model. The expression of selected cytokines and chemokines in the ear tissues were studied by immunohistochemical staining conducted 24 h after croton oil application. TNF-α, IL-1β, IL-6, chemokine (C-X-C motif) ligand (CXCL)-1, macrophage inhibitory factor (MIF), and monocyte chemoattractant protein (MCP)-1 were strongly induced and diffusely expressed in epidermis and dermis in the control animals. In contrast, that expression of these cytokines was all strongly suppressed by the topical application of rhTRX (Figure 2A). We also determined the mRNA expression of these cytokines by real-time RT-PCR 24 h after croton oil treatment. We used RNA extracted from the skin of the back of the mice, because the amount of RNA that can be extracted from the skin of the ears is so low that it cannot be used for quantitative analysis. The mRNA expression of TNF-α, IL-1β, IL-6, CXCL-1, and MCP-1 were significantly suppressed in the TRX treated group, compared to the BSA treated group (**P < 0.01, *P < 0.05) (Figure 2B). These results suggested that topically applied rhTRX suppressed ICD by inhibiting the production of the inflammatory cytokines and chemokines.

### Pharmacokinetics of topically applied rhTRX

**Figure 3:**
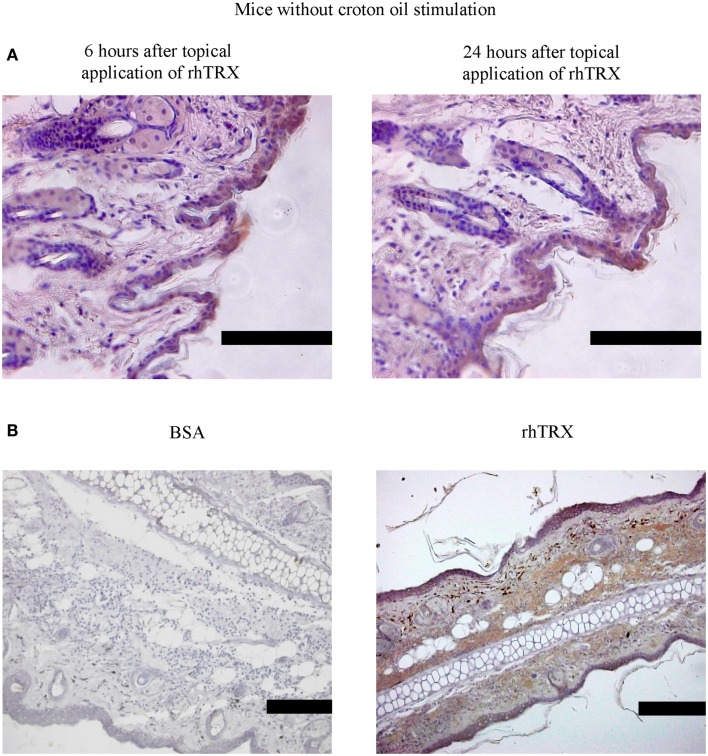
**rhTRX was distributed only in the epidermis in mice without croton oil stimulation, but it was distributed in both the epidermis and dermis in irritant dermatitis**. **(A)** rhTRX was only distributed in the epidermis and cutaneous appendages at 6 and 24 h in the mice without the croton oil stimulation. Bars, 100 μm (*n* = 5). **(B)** After croton oil was applied, the rhTRX was distributed in both the epidermis and dermis in irritant dermatitis in the group with the rhTRX pre-treatment at 24 h. Bars, 50 μm (*n* = 5).

Next we examined the distribution of topically applied rhTRX in murine skin tissues under non-inflammatory and inflammatory conditions. Twenty micrograms per milliliter rhTRX was applied on the surface of the murine ears, and ear specimens were stained with anti-human monoclonal TRX antibody. The anti-TRX antibodies do not cross-react with the mouse TRX, verifying that only exogenously applied rhTRX was detected in the histological analysis. rhTRX was distributed in the epidermis and cutaneous appendages under non-inflammatory conditions (Figure 3A). In contrast, rhTRX was distributed in both the epidermis and the dermis under inflammatory condition (Figure 3B). No rhTRX was detected by ELISA in the blood or the urine 6 and 24 h after it was applied under either non-inflammatory or inflammatory conditions (data not shown). These findings indicated that topically applied rhTRX penetrates into skin tissue to exert its anti-inflammatory effect on ICD, but it does not diffuse into the circulation.

### rhTRX suppressed croton oil-induced expression of TNF-α, IL-1β, IL-6, CXCL-1, and MCP-1 mRNA in cultured murine keratinocytes

As shown in Figure 2, the cytokines were diffusely expressed in the epidermis, suggesting that the cytokines were possibly produced by keratinocyte in the ICD mice model. Therefore we selected murine keratinocytes (PAM 212 cells), as the target to detect the effect of TRX on the production of cytokines after croton oil treatment.

**Figure 4:**
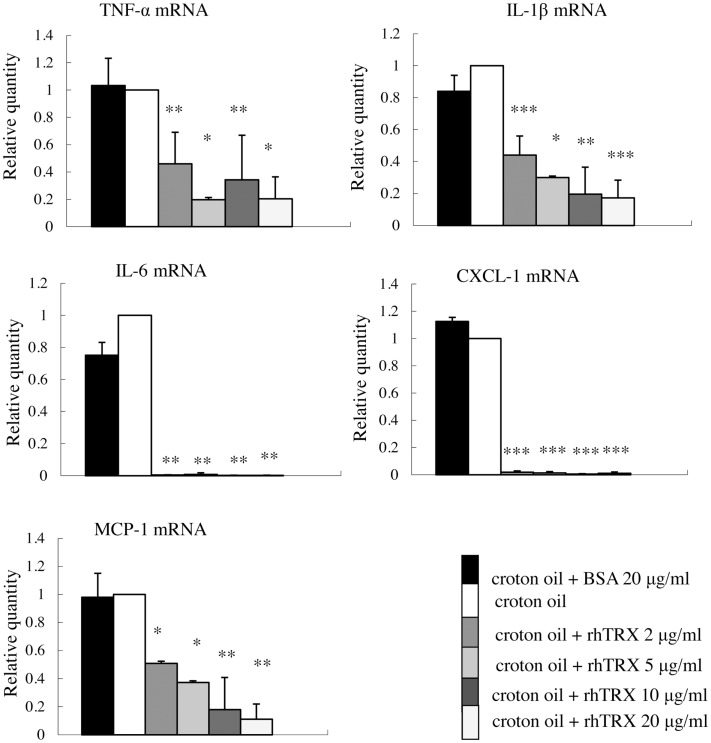
**The application of rhTRX significantly suppressed the mRNA expression of TNF-α, IL-1β, IL-6, CXCL-1, and MCP-1 in PAM 212 cells stimulated by croton oil**. Croton oil was added to the culture medium in the presence or absence of rhTRX (2–20 μg/ml). The mRNA expression of TNF-α, IL-1β, IL-6, CXCL-1, and MCP-1 were measured by real-time RT-PCR 24 h after stimulation. The mRNA expression in the rhTRX treatment group was significantly suppressed by 2–20 μg/ml rhTRX, compared with the BSA treatment groups. The mRNA expression levels were determined relative to croton oil-stimulated cells. Values are shown as the mean ± SD of three experiments (*P < 0.05, **P < 0.01, ***P < 0.001) (Student *t*-test).

**Figure 5:**
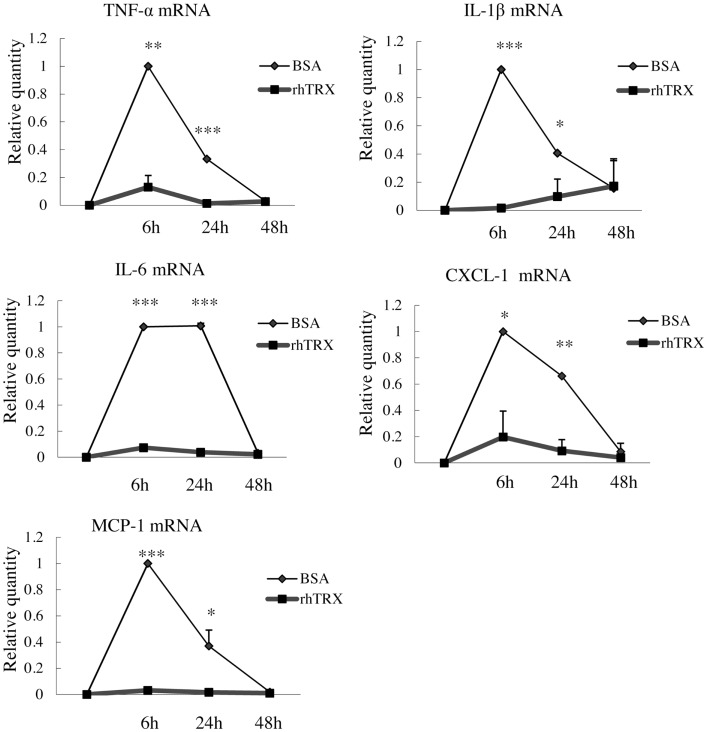
**The application of rhTRX can suppress the mRNA expression of cytokines and chemokines in PAM 212 cells after croton oil stimulation for at least 24 h**. After croton oil stimulation, the mRNA expression of TNF-α, IL-1β, IL-6, CXCL-1, and MCP-1 was measured by real-time RT-PCR at the indicated time points. Application of 10 μg/ml rhTRX significantly suppressed the mRNA expression of the cytokines and the chemokines at 6 and 24 h after the croton oil was applied. The mRNA expression levels were determined relative to croton oil-stimulated cells. Values are shown as the mean ± SD of three experiments (*P < 0.05, **P < 0.01, ***P < 0.001) (Student *t*-test).

In order to investigate the optimal concentration of croton oil which induces the production of cytokines by PAM 212 cells, the cell viability was assessed by a lactate dehydrogenase (LDH) release assay 24 h after croton oil treatment. The application of 2–60 μg/ml croton oil did not cause cell damage (data not shown). Therefore, 20 μg/ml croton oil was selected for subsequent experiments. The cell lines were incubated with varying amounts of rhTRX immediately after croton oil treatment. The mRNA expression of TNF-α, IL-1β, IL-6, CXCL-1, and MCP-1 was determined by real-time RT-PCR 24 h after the addition of croton oil. The results indicate that the expression of the cytokines and chemokines was significantly suppressed by the addition of 2–20 μg/ml rhTRX. *P* values in 2–20 μg/ml rhTRX treatment groups are shown as follows: (***P < 0.001, **P < 0.01, *P < 0.05) (Figure 4). In order to detect the time course of the effects of TRX, 10 μg/ml rhTRX was added to the culture medium, immediately after 20 μg/ml croton oil was applied, and incubated for 6, 24, and 48 h, respectively. In control cells, the expression levels of these cytokines were increased within 6 h in response to croton oil, and returned to the basal levels at 48 h post stimulation. Elevation of these inflammatory cytokines was significantly suppressed in the presence of rhTRX. *P* values in the groups which treat with rhTRX 6 and 24 h later are shown as follows: (***P < 0.001, **P < 0.01, *P < 0.05) (Figure 5). These findings indicate that rhTRX suppresses the mRNA expression of cytokines produced by keratinocytes.

### rhTRX suppressed the release of TNF-α, IL-6, and MCP-1 by murine keratinocytes, after stimulation with croton oil

**Figure 6:**
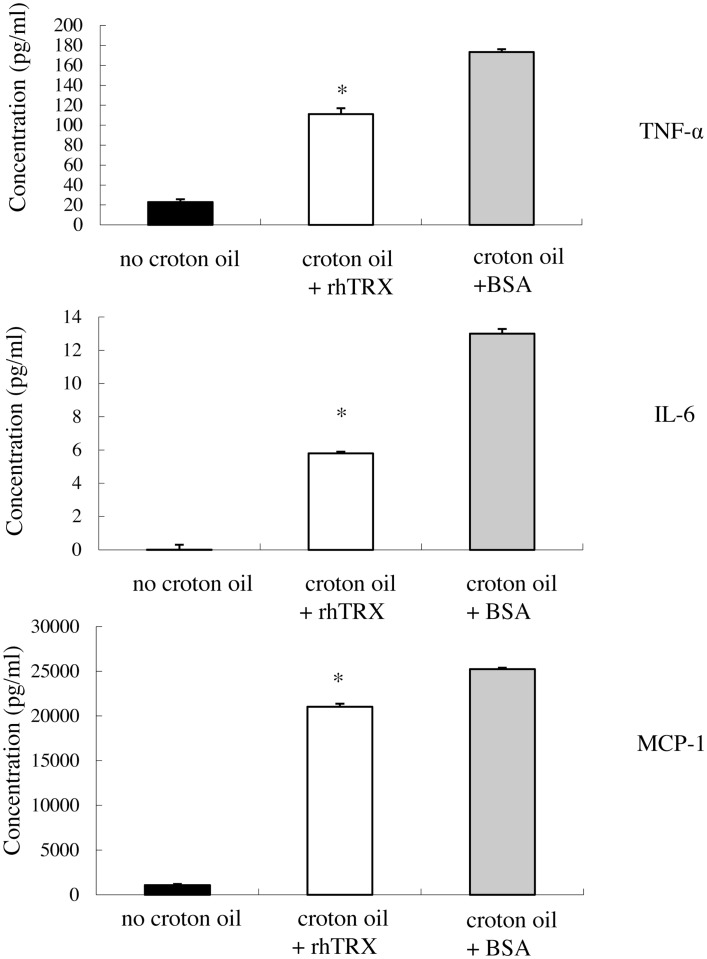
**After stimulation with croton oil, rhTRX suppressed the release of TNF-α, IL-6, and MCP-1 by PAM 212 cells into the medium**. PAM 212 cells were left untreated (no croton oil) or stimulated with croton oil in the presence of BSA or rhTRX (10 μg/ml). The concentrations of TNF-α, IL-6, and MCP-1 in the culture supernatant were measured by CBA assay 24 h after croton oil treatment. The concentrations of TNF-α, IL-6, and MCP-1 were significantly lower than those shown in the BSA application group (*P < 0.05). Values are shown as the mean ± SD of the samples (Student *t*-test).

In order to investigate whether rhTRX suppresses the release of the cytokines from PAM 212 cell into culture medium, 10 μg/ml rhTRX was added to the culture medium immediately after the addition of croton oil, and a Cytometric Bead Array analysis was conducted to quantify the concentrations of TNF-α, IL-6, and MCP-1 in the culture supernatant 24 h after the addition of croton oil. We have demonstrated that the concentration of applied croton oil did not affect the cell viability. The accumulation of TNF-α, IL-6, and MCP-1 in the culture supernatant was significantly suppressed by the application of rhTRX, compared to BSA (*P < 0.05) (Figure 6). These results indicate that rhTRX could act on keratinocytes to prevent the production and release of cytokines induced by croton oil.

### PMA-induced TNF-α production was suppressed by rhTRX in cultured murine keratinocytes

**Figure 7:**
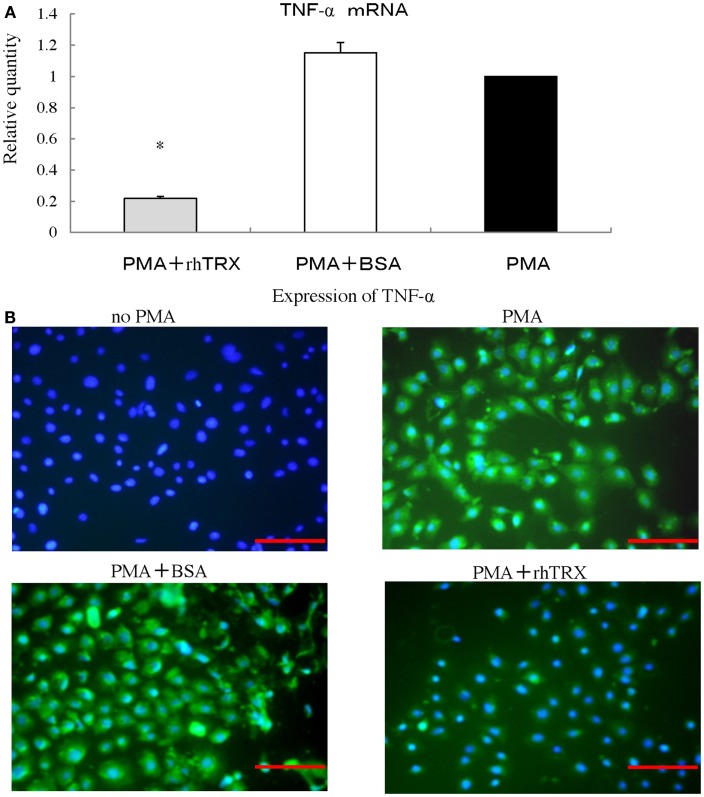
**After stimulation with phorbol 12-myristate 13-acetate (PMA), rhTRX suppressed the expression of TNF-α produced by murine keratinocytes**. **(A)** Ten nanomolar PMA was added to the medium, and cells were incubated for 6 h. The mRNA expression levels of TNF-α were determined by real-time RT-PCR. The TNF-α mRNA expression was significantly suppressed by 10 μg/ml rhTRX (*P < 0.05). The mRNA expression levels were determined relative to PMA-stimulated cells. Values are shown as the mean ± SD of two experiments (Student *t*-test). **(B)** The expression of TNF-α in the cells was detected by immunocytochemistry 24 h after simulation. The application of 10 μg/ml rhTRX significantly suppressed the expression of TNF-α produced by PAM 212 cells stimulated by PMA (expression of TNF-α, FITC, green; nuclear staining, DAPI, blue). Bars, 100 μm.

We investigated the effect of rhTRX on the PMA-induced production of TNF-α in PAM 212 cells. Ten micrograms per milliliter rhTRX was added immediately after PMA treatment, and then incubated for 6 h for real-time RT-PCR analysis. The mRNA expression of TNF-α in the rhTRX treatment group was significantly suppressed, compared with the BSA treatment group (*P < 0.05) (Figure 7A). The cells were also incubated for 24 h for an immunocytochemical study, because the production of protein is slower than the production of mRNA. TNF-α was strongly expressed in the PMA and PMA + BSA treatment groups, whereas the expression of TNF-α was strongly inhibited by the rhTRX application (Figure 7B). These findings clearly showed that cytokines are produced by keratinocytes, and suppressed by rhTRX application.

## Discussion

Functioning as intercellular mediators, cytokines are produced by lymphocytes as well as by effector cells during inflammatory responses. Particularly, IL-1 and TNF-α have been implicated in many diseases including ICD, which accounts for 20–80% of all cases of contact dermatitis depending on the country (16, 17). In addition, as an immune mechanism in the pathogenesis of ICD, it has become quite clear that exposure to various irritant exerts toxic effects on keratinocytes, activating innate immunity with the release of TNF-α, IL-1β, IL-6, and IL-8 from keratinocytes (17–19). In turn, the cytokines activate Langerhans cells, dermal dendritic cells, and endothelial cells, all of which contribute to cellular recruitment to the site of the keratinocyte damage. In ICD as well as ACD, infiltrating cells include neutrophils, lymphocytes, macrophages, and mast cells, which further promote the inflammatory cascade (17–19). These data strongly suggest that keratinocytes play a major role in the pathogenesis of ICD. In this study, we used the well-established model of ICD induced by croton oil, of which major active component is PMA (15). It has been reported that topically applied croton oil or PMA-induced production of cytokines and chemokines such as TNF-α, IL-1β, IL-6, and CXCL-1 in murine skin (13, 20). We also demonstrated that not only TNF-α, IL-1β, IL-6, and CXCL-1, but also MCP-1 were highly induced in the region of skin epidermis mainly composed of keratinocytes after treatment with croton oil (Figure 2). Furthermore, both our *in vitro* study and the previous study showed that the cytokines and chemokines were produced by the keratinocytes stimulated with croton oil or PMA. Although our results would not exclude possible involvement of cellular factors other than keratinocytes, our results, together with previous studies in the literature (13, 21, 22), strongly suggest that keratinocytes are the primary source of the inflammatory mediators in the murine ICD model.

Several explanations of the anti-inflammatory effects of TRX have been reported: (a) anti-oxidant effect (2), (b) anti-leukocyte chemotaxis (3), (c) suppression of neutrophil adhesion on endothelial cells through membrane TRX-1 (5), (d) suppression of complement activation, and (e) inhibition of the MIF (23, 24). In this study, we demonstrated that topically applied rhTRX suppressed the production of inflammatory cytokines and chemokines, which would more likely explain the protective effects of TRX under inflammatory conditions, in addition to anti-leukocyte chemotaxis. Previous reports have shown that exogenous TRX suppresses the production of cytokines and chemokines in some diseases, such as myosin-induced autoimmune myocarditis (7), indomethacin-induced gastric injury (9), and influenza A virus induced acute lung injury (25). These findings also strongly support the concept that attenuation of cytokine production is one of the mechanisms underlying the anti-inflammatory effect of TRX. It has been reported that exogenously administered human TRX suppresses lipopolysaccharide-induced neutrophil recruitment (23). We also demonstrated that topical application of rhTRX suppressed the infiltration of neutrophils to dermal tissues, where the chemotactic factors such as CXCL-1 and MCP-1 were decreased (Figure 2A). We suppose that rhTRX inhibited the production of these chemokines by keratinocytes in inflammatory disorders such as ICD and ACD, thereby suppressing the neutrophils chemotaxis. Taken together, all of these findings suggest that exogenous TRX suppresses inflammatory reactions by inhibiting the production of cytokines and chemokines. In addition to croton oil and PMA, we also used polyriboinosinic-polyribocytidilic acid (polyI: C), an immunostimulant known to interact with toll-like receptor (TLR) 3, to investigate the anti-inflammatory effects of TRX. TRX suppressed the production of IL-33 and CXCL-1 by PAM 212 cells induced by polyI: C (data not shown), indicating that virus-associated immune responses in keratinocytes, as well as PMA-induced inflammation, can be regulated by TRX applied exogenously.

It is unclear how extracellular TRX regulates inflammatory processes. Accumulating evidence indicates that oxidative stress is associated with inflammation, and thiol anti-oxidants, especially glutathione, have often been thought of as possible anti-inflammatory mediator (26). Indeed, oxidative stress plays an important role in many inflammatory diseases, including ICD (27–29). A molecular mechanism for this association was first provided in 1991, with the finding that H_2_O_2_ activates the transcription factor NF-κB, which is involved in the production of many inflammatory cytokines, while thiol anti-oxidants inhibit its activation (30). Several studies have reported inhibition of cytokine production by many thiol anti-oxidants. As a redox regulatory protein, TRX can scavenge ROS, either directly or in cooperation with peroxiredoxin (24). Given that oxidative stress is induced by treatment with croton oil in murine skin (31, 32), TRX might reduce the cellular oxidative stress under inflammatory conditions, which would contribute to the suppression of the ROS dependent activation of inflammatory signaling. Actually, it has been reported that extracellular TRX inhibited LPS-induced activation of the NF-κB pathway in cultured macrophages (33), which could be involved in the down-regulation of inflammatory cytokines, including IL-1β, IL-6, IL-8, and TNF-α. Alternatively, extracellular TRX may suppress the activation of inflammatory signaling by acting on cell surface molecules to control their function through its reduction activity. Exogenously applied TRX was associated with lipid rafts (34), which are specialized membrane domains enriched in cholesterol and glycosphingolipids (35). Since lipid rafts serve as a membrane platform for the assembly of signaling complexes, extracellular TRX may interact with the components of lipid rafts and modulate the redox properties on the cell surface, potentially leading to dynamic changes in the cellular response to inflammatory stimuli.

It was also suggested that extracellular thioredoxin could be transported into cells through membrane lipid rafts (34, 36), and control intracellular redox balance. As an endogenous negative regulator of thioredoxin, thioredoxin-binding protein-2 (TBP-2) directly binds thioredoxin to inhibit thioredoxin-reducing activity (37). We propose a redox-sensitive signaling complex named redoxisome (38), comprising TRX and TBP-2, which regulates a great variety of redox-sensitive signals. Thus we suppose that the redox-dependent signalosome may be involved in the regulation of inflammatory pathway. It was reported that TBP-2 deficiency promotes TNF-α-induced NF-κB activity (39). TBP-2^-/-^ mice injected with LPS did not show higher serum levels of TNF-α, IL-6, IL-10, Interferon (IFN)-β, IFN-γ, MCP-1, or macrophage inflammatory protein-2 (MIP-2), compared with wild-type mice (40). As our next strategic aim, we will conduct a study on the molecular mechanism of extracellular TRX for suppressing cytokine production.

Here we demonstrated that the topical application of rhTRX is very effective for treatment of ICD by suppressing the production of cytokines and chemokines, and topically applied rhTRX was distributed limitedly in the skin, but not the blood or the urine. Because topical application would be more practical and safe in clinical medicine, compared to internal administration or injections, this finding was quite meaningful and novel. Our study also showed that both pre- and post-treatment with rhTRX significantly suppressed ICD response, indicating that the topical application of rhTRX can have a promising effect on not only prophylactic but also therapeutic medicine. In addition, our results suggest that rhTRX has a direct effect on keratinocytes widely distributed throughout the dermatitis lesion. Taken together, these findings suggest that TRX could be a useful, near ideal agent for the treatment of dermatitis.

In conclusion, we demonstrated that the topical application of rhTRX suppressed ICD by inhibiting the production and release of cytokines and chemokines. One might expect that TRX is useful for the treatment of a variety of skin and mucosal inflammatory disorders.

## Materials and Methods

### Mice

Wild-type female C57BL6 mice (8 weeks old) were purchased from Charles River Japan (Tokyo, Japan). All animals were maintained in microisolator cages and exposed to a 12-h light/12 h dark cycle, with standard feed and water *ad libitum*. All experiments were conducted according to the Institutional guidelines and regulations.

### ICD model

In order to induce ICD, 10 μl of 2% croton oil (Sigma, St. Louis, MO, USA) dissolved in acetone/olive oil (4:1) was applied to the dorsal and ventral aspects of both sides of the murine ears and back. Ear swelling was measured in a blinded fashion with a digimatic micrometer (Mitutoyo, Tokyo, Japan), 6 or 24 h after the croton oil treatment. The mice were euthanized immediately after the experiment was concluded, and the target tissue samples were removed.

### Topical application of rhTRX

Twenty micrograms per milliliter rhTRX in phosphate-buffered saline (PBS) (Redox Bio Science, Japan) was topically applied by the nano-spray machine (quantity of mist: 1 ml/min) on the dorsal and ventral aspects of the ears and the back (5 s, distance 10 cm) (Konishi Seiko Japan), and 20 μg/ml of BSA was applied as a control. rhTRX was denatured at 100°C for 20 min. About 1.7 μg of rhTRX was applied to the entire surface of mouse ear (about 1 cm^2^).

### Histological analysis of ICD

The ears were fixed in formalin for 24 h, embedded in paraffin, and stained with hematoxylin and eosin. The numbers of neutrophils in the dermis were counted for a quantitative analysis in 10 microscopic fields of 5 different specimens, and the average number of neutrophils was obtained. The caliber of the capillary blood vessels was measured as a quantitative indication of the dilatation of the capillary blood vessels. The caliber sizes of 20 randomized vessels in the dermis and the subcutaneous tissues from both the TRX and BSA treatment groups were measured by microscope, in order to obtain the average size.

### Immunohistochemistry

Briefly, tissue samples were prepared as 3 μm-thick sections from paraffin-embedded specimens, followed by deparaffinization and blocking of endogenous peroxidase activity with 3% hydrogen peroxide in methanol for 15 min, and 10% bovine serum was added for 30 min to block non-specific binding at room temperature. The tissue specimens were incubated with one of the rabbit anti-mouse TNF-α polyclonal antibody (Hycult Biotech), goat anti-mouse IL-1β antibody (R and D systems), goat anti-mouse IL-6 polyclonal antibody (Santa Cruz Biotechnology), goat anti-mouse CXCL-1 polyclonal antibody (R and D Systems), anti-MIF polyclonal antibody (Invitrogen), rat anti-mouse MCP-1 monoclonal antibody (Hycult Biotech), and mouse anti-human monoclonal TRX antibody (Redox Bio Science) as primary antibodies overnight at 4°C. After being washed with PBS, sections were incubated with a biotinylated anti-rabbit immunoglobulin for TNF-α (Dako), biotinylated anti-goat immunoglobulin for IL-1β, IL-6, and CXCL-1 (Dako), biotinylated anti-rat immunoglobulin for MIF and MCP-1 (Dako) for 30 min, followed by streptavidin-conjugated horseradish peroxidase (Dako) for 30 min at room temperature. The specimens, incubated with mouse anti-human TRX monoclonal antibody, were treated at room temperature with a Histofine^®^ mouse stain kit (Nichirei Corporation) to block non-specific binding, then with 3,3′-diaminobenzidine working solution (Vector Laboratories, Burlingame CA, USA).

### Cell culture and cell treatment

A spontaneously transformed BALB/c keratinocyte cell line, PAM 212, obtained from Dr. Steve Uilric (Department of Immunology, MD Anderson Cancer Center, Houston, TX, USA), was used in the study. Cells were maintained in RPMI 1640 (Sigma chemical) supplemented with heat-inactivated 10% FBS (Hyclone), Hepes (10 mM) (Sigma), 1% non-essential amino acids (Sigma), L-glutamine (2 mM), sodium pyruvate (1 mM) (Sigma), 100 U/ml penicillin (Sigma), 100 μg/ml streptomucin (Sigma), 0.25 μg/ml amphotericin B (Sigma), and 2-mercaptoethanol (Sigma).

Cells were cultured at 37°C in 95% air/5% CO_2_. PAM 212 cells in passage 1–3 were seeded into six well plates at a density of 2 × 10^5^/2 ml medium per well and cultured for 24 h. Twenty micrograms per milliliter croton oil/0.1% ethanol (13) or 10 nM PMA (41) was added to the medium in the presence or absence of rhTRX (0–20 μg/ml).

### Real-time RT-PCR analysis

Total RNA was extracted using a QuickGene RNA tissue kit SII (Fujifilm, Tokyo, Japan) for the tissue, and a QuickGene RNA cultured cell kit SII (Fujifilm, Tokyo, Japan) for the cells. Total RNA was reverse transcribed using the PrimeScript RT Reagent Kit (Takara, Shiga, Japan). A real-time PCR was performed using the SYBR^®^ Premix Ex Taq™ II (Takara, Shiga, Japan). All primers were purchased from Takara Bio, and Glyceraldehyde-3-phosphate dehydrogenase (GAPDH) was used as the housekeeping gene. The reaction was performed with ABI PRISM 7500 Sequence Detection System (Applied Bio Systems, Tokyo, Japan) to quantify the mRNA, according to the manufacturer’s protocol.

### Cytometric bead array analysis

The concentration of IL-6, MCP-1, and TNF-α in the culture supernatant of the PAM 212 cells was measured using the BD Cytometric Bead Array mouse inflammation kit (BD Biosciences), following the manufacturer’s protocol.

### Immunocytochemistry

The samples were fixed in cold methanol for 20 min, and incubated for 10 min with PBS containing 0.25% Triton X-100 for permeabilization. One percent BSA in PBS was added for 30 min to block unspecific binding of the antibodies. The samples were incubated with rabbit anti-mouse TNF-α antibody (Hycult Biotech) overnight at 4°C. Biotinylated anti-rabbit immunoglobulin (Dako) was added for 1 h at room temperature. Streptavidin/FITC (Dako) was added for 30 min in the dark, the samples were then incubated with 1 μg/ml DAPI for 1 min for DNA staining (Invitrogen).

### Statistical analysis

Results were expressed as mean ± SD. After the data were tested for normal distribution using the Shapiro–Wilk test, the normal distributed data were evaluated by the Student *t*-test for comparisons between two groups, and the non-normal distributed data was tested by Wilcoxon signed-rank test. Findings of *P* < 0.05 were considered statistically significant.

## Conflict of Interest Statement

The authors declare that the research was conducted in the absence of any commercial or financial relationships that could be construed as a potential conflict of interest.
